# Autologous Tissue Repair and Total Face Restoration

**DOI:** 10.1001/jamaoto.2024.1572

**Published:** 2024-07-03

**Authors:** Tao Zan, Wenjin Wang, Haizhou Li, Caiyue Liu, Hainan Zhu, Yun Xie, Shuangbai Zhou, Yashan Gao, Xin Huang, Shuchen Gu, Kai Liu, Bin Gu, Feng Xie, Lee L. Q. Pu, Qingfeng Li

**Affiliations:** 1 Department of Plastic and Reconstructive Surgery, Shanghai Ninth People’s Hospital, Shanghai Jiao Tong University, School of Medicine, Shanghai, China; 2 Division of Plastic Surgery, University of California, Davis, Sacramento

## Abstract

**Importance:**

Total face restoration remains a challenge in modern reconstructive surgery. After 17 years of experiments and preliminary clinical studies, a new concept of face prefabrication was developed for face restoration with autologous tissue.

**Objective:**

To evaluate the long-term results of face restoration with autologous tissue and report a finalized and standardized approach of face prefabrication.

**Design, Setting, and Participants:**

In this single-center long-term retrospective study, 32 patients who underwent total face restoration between 2005 and 2022 were reviewed. These patients underwent total facial reconstruction, which included flap prefabrication, 3-dimensional printing, tissue expansion, and flap transfer with aid of indocyanine green angiography (IGA). The flap first undergoes prefabrication by transferring vascularized fascia under the skin of the selected chest. A tissue expander is then placed under the fascia to create a large, thin, reliable skin flap after expansion. Once completed, the flap is transferred to the face during the second stage of the reconstruction. Intraoperative IGA is performed to guide the design of subsequent openings for facial fissures. Data were analyzed from July to September 2023.

**Main Outcomes and Measures:**

Flap healing, reconstructive outcome, and patient recovery were assessed during follow-up. Three questionnaires, including the 36-Item Short Form Health Survey (SF-36), Aesthetic and Functional Status Score of Facial Soft-Tissue Deformities/Defects, and the EuroQoL Health-Related Quality of Life (EQ-5D-5L), were used to evaluate the quality of life and satisfaction with facial aesthetic and functional status.

**Results:**

Of 24 included patients, 14 (58%) were male, and the mean (range) age was 32.9 (8-62) years. The mean (range) follow-up was 5.6 (2-12) years. All patients reported a significant improvement in quality of life (SF-36), especially in mean (SD) social functioning (preoperative score, 53.65 [34.51]; postoperative score, 80.73 [19.10]) and emotional stability (preoperative score, 56.67 [25.55]; postoperative score, 71.17 [18.51]). A total of 22 patients (92%) went back to work. Mean (SD) facial aesthetic status (preoperative score, 4.96 [3.26]; postoperative score, 11.52 [3.49]; *P* < .001) and functional status (preoperative score, 11.09 [3.51]; postoperative score, 15.78 [3.26]; *P* < .001) also improved. In addition, there was a significant increase in overall satisfaction and self-reported health status (preoperative score, 8.13 [1.52]; postoperative score, 3.58 [2.31]).

**Conclusions and Relevance:**

In this study, 5-year follow-up results suggested that this innovative approach to total face restoration offered a safe and valid option for indicated patients, with acceptable reconstructive and cosmetic outcomes.

## Key Points

**Question:**

What are the long-term outcomes of total face restoration with autologous tissue using skin expansion and flap prefabrication assisted with 3-dimensional printing and indocyanine green–assisted monitoring?

**Findings:**

In this cohort study including 24 patients, patients showed improved facial aesthetic and functional status and reported a significant improvement in quality of life and in overall satisfaction and self-reported health status up to 12 years after treatment (mean, 5 years’ follow-up).

**Meaning:**

In this study, incorporation of both conventional and innovative approaches, such as flap prefabrication, 3-dimensional printing, and indocyanine green–assisted monitoring, significantly broadened the capabilities of autologous tissue reconstruction in facial restoration and yielded superior outcomes.

## Teaser

This cohort study evaluates the long-term results of face restoration with autologous tissue using skin expansion and flap prefabrication assisted with 3-dimensional printing and indocyanine green–assisted monitoring.

## Introduction

Severe facial disfigurement not only results in a profound loss of identity but also leads to what can be described as social death, a condition that is both physically and psychologically devastating.^1^ Facial reconstruction, therefore, carries the immense expectation of transforming the lives of these patients and restoring their fundamental human rights. Despite this, the complex nature of facial restoration continues to pose one of the most formidable challenges in modern medicine.

Facial restoration demands not just total facial coverage but also the meticulous reconstruction of delicate facial features. Traditional approaches, ranging from skin grafts to free flaps, have been extensively explored. However, these techniques frequently fall short, primarily due to mismatches in size, color, and texture, leading to a patchwork appearance and conspicuous scarring on the face. The bulkiness of standard flaps often obscures the subtle contours and emotional expressions of the face.^2,3,4^ Consequently, these methods have historically been seen as a last resort for wound closure, offering limited success.^5^

In recent years, face allotransplant has emerged as a promising solution for severe disfigurements involving composite facial tissues.^6,7,8,9,10^ Despite its potential, the clinical application of face allotransplant remains restricted, largely due to the requirement of lifelong immunosuppression and its associated long-term risks.^11,12,13,14^

Over the past 17 years, our team has dedicatedly pursued extending the capabilities of autologous tissue in total face restoration. This journey encompassed a multitude of experimental and preliminary clinical studies.^15,16,17,18,19^ With a cumulative 17 years of experience in both clinical practice and basic science, we have now successfully integrated various technical elements into a coherent, systematic approach. This approach involves a series of standardized procedures specifically designed for comprehensive facial reconstruction. Our method distinguishes itself from conventional autologous tissue repair techniques by transcending mere wound coverage. It strategically facilitates the reconstruction of intricate facial features while maximally preserving facial expressions. Our innovative method comprises flap prefabrication, 3-dimensional printing, skin and soft-tissue expansion, and flap transfer, with aid of indocyanine green (ICG) angiography (IGA) to ensure optimal outcomes. In this article, we present our refined approach, assessing its effectiveness and durability through a long-term follow-up study.

## Methods

All patients provided written informed consent to participate in the clinical trial, as approved by the ethics committee of Shanghai Ninth People’s Hospital, Shanghai Jiao Tong University School of Medicine (SH9H-2020-T8-2). This study was registered on ClinicalTrials.gov (NCT04405687). This study followed the Strengthening the Reporting of Observational Studies in Epidemiology (STROBE) reporting guideline. A total of 32 patients with total face deformities were enrolled in this retrospective study.

### Inclusion and Exclusion Criteria

The patients enrolled in this study experienced total facial deformities involving all the facial units, a subset of type IV facial deformities, as we previously reported.^19^ All patients were 6 years or older and able to comply with postoperative care and follow-up examinations. Patients were excluded if they were unable to provide informed consent or did not show up for follow-up visits.

### Study Design and Participants

The initial screening included medical record review of history, classification of facial deformities, and collection of demographic data. If patients were determined eligible and signed the informed consent willingly, preoperative and postoperative photographs were taken and analyzed. All patients had face restoration with autologous tissue. At each follow-up visit, data collected included physical examination characteristics, photographs, and adverse event reporting. The patients and their family members were informed of all known risks and benefits of the treatment plan.

For preoperative planning, 3-dimensional simulation was performed to define the defects involved with regards to both the soft tissues and the underlying supportive structures. Photographs were taken before, during, and after treatment.

### Surgical Procedures

**Figure 1.**
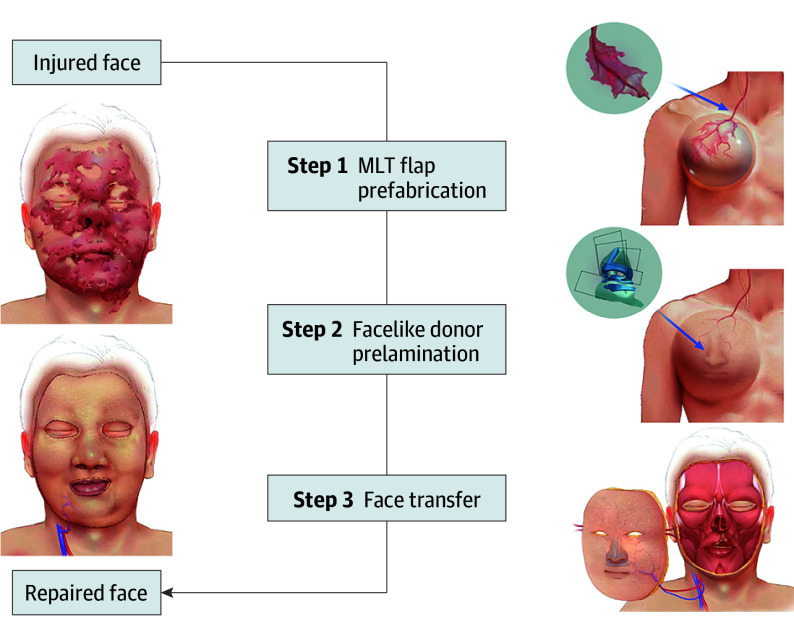
Schematic Diagram Demonstrating Innovative Approach to Total Facial Reconstruction The total facial reconstruction procedure involved 3 steps. The first step involves prefabrication and tissue expansion by harvesting the descending branch of the lateral circumflex femoral vessels and surrounding fascia as a free fascial flap and placing it in the subcutaneous pocket of the donor site before the placement of the tissue expander, which is placed underneath the fascial flap. The second step involves prelamination in which the nose and lips formation are forged by a 3-dimensional print-assisted cartilage framework implant. The third step involves face transfer and flap fenestration in which the expanded and prefabricated flap are transfered to the face both as a pedicled flap and supercharged by 2 additional anastomoses of vascular pedicles. Intraoperative indocyanine green angiography was applied to evaluate flap perfusion and to assist in guiding subsequent openings for the mouth, nostrils, and palpebral fissures. MLT indicates the Match, Large, Thin properties.^20^

The surgical procedure is composed of 3 parts (Figure 1). The first step involves prefabrication and tissue expansion by harvesting the descending branch of the lateral circumflex femoral vessels and surrounding fascia as a free fascial flap and placing it in the subcutaneous pocket of the donor site before placement of the tissue expander, which is placed underneath the fascial flap (eMethods and eFigure 1 in Supplement 1).^18^ The second step involves prelamination in which the nose and lips formation are constructed by a 3-dimensional print-assisted cartilage framework implant.^21^ The third step involves face transfer and flap fenestration in which the expanded and prefabricated flap are transferred to the face both as a pedicled flap and supercharged by 2 additional anastomosis of vascular pedicles. Intraoperative IGA was applied to evaluate flap perfusion and to assist in guiding subsequent openings for the mouth, nostrils, and palpebral fissures.^22^

### Follow-Up Assessments

All patients answered all questionnaires. Three questionnaires, including the 36-Item Short Form Health Survey (SF-36), Aesthetic and Functional Status Score of Facial Soft-Tissue Deformities/Defects, and the EuroQoL Health-Related Quality of Life (EQ-5D-5L), were assessed based on preoperative and postoperative situations on different days to avoid interactions (eAppendixes 1, 2, and 3 in Supplement 1). Facial expression function was evaluated by photograph and video documentation by 3 independent physicians (W. W., X. H., and S. G.). Facial expression functions were classified into 3 degrees: grade 1 indicated basic occlusion function, including the orbicularis oris and ocular muscles; grade 2, basic social expression, including basic facial expressions showing happiness and sadness; and grade 3, delicate facial expressions, including facial expressions showing happiness and sadness to different degrees.

### Statistical Analysis

The raw scale scores of the SF-36 in 9 aspects, including physical functioning, role physical, bodily pain, general health, vitality, social functioning, role emotional, mental health, and reported health transition, were calculated and transformed to a scale ranging from 0 to 100 according to its manual and interpretation guide. In Aesthetic and Functional Status Score of Facial Soft-Tissue Deformities/Defects, each aspect was valued from 0 to 3, and the scores of aesthetic and functional statuses were summed (aesthetic status score range, 18 points; functional status score range, 21 points) for further analysis.

All data are expressed as means with SDs and analyzed using SPSS software version 21 (IBM). The preoperative and postoperative data were compared with a paired *t* test, and *P* values less than .05 were considered statistically significant. The comparison between the 2 groups was conducted using a 2-tailed unpaired *t *test if the data fit a normal distribution and variances were similar by *F* test. Data were analyzed from July to September 2023.

## Results

### Patients

From 2005 to 2022, 32 patients who had total face disfigurement were repaired with our face prefabrication technique in our center. The retrospective medical record review was performed on all patients. Of 24 included patients, 14 (58%) were male, and the mean (range) age was 32.9 (8-62) years. A total of 8 patients were excluded in the final analysis as they did not show up for follow-up visits. The mean (range) follow-up was 5.6 (2-12) years. The causes of injuries included flame burns in 20 patients and chemical burns in 4 patients. The size of tissue flaps ranged from 15 × 16 cm^2^ to 38 × 34 cm^2^.

Major complications were partial graft necrosis in 2 patients. One patient had a 5 × 3–cm necrosis on his left temporal region, which healed by itself. Another patient had 8 × 5–cm necrosis on the left frontal area, which was repaired by secondary skin grafting.

#### Aesthetic Outcome

**Figure 2.**
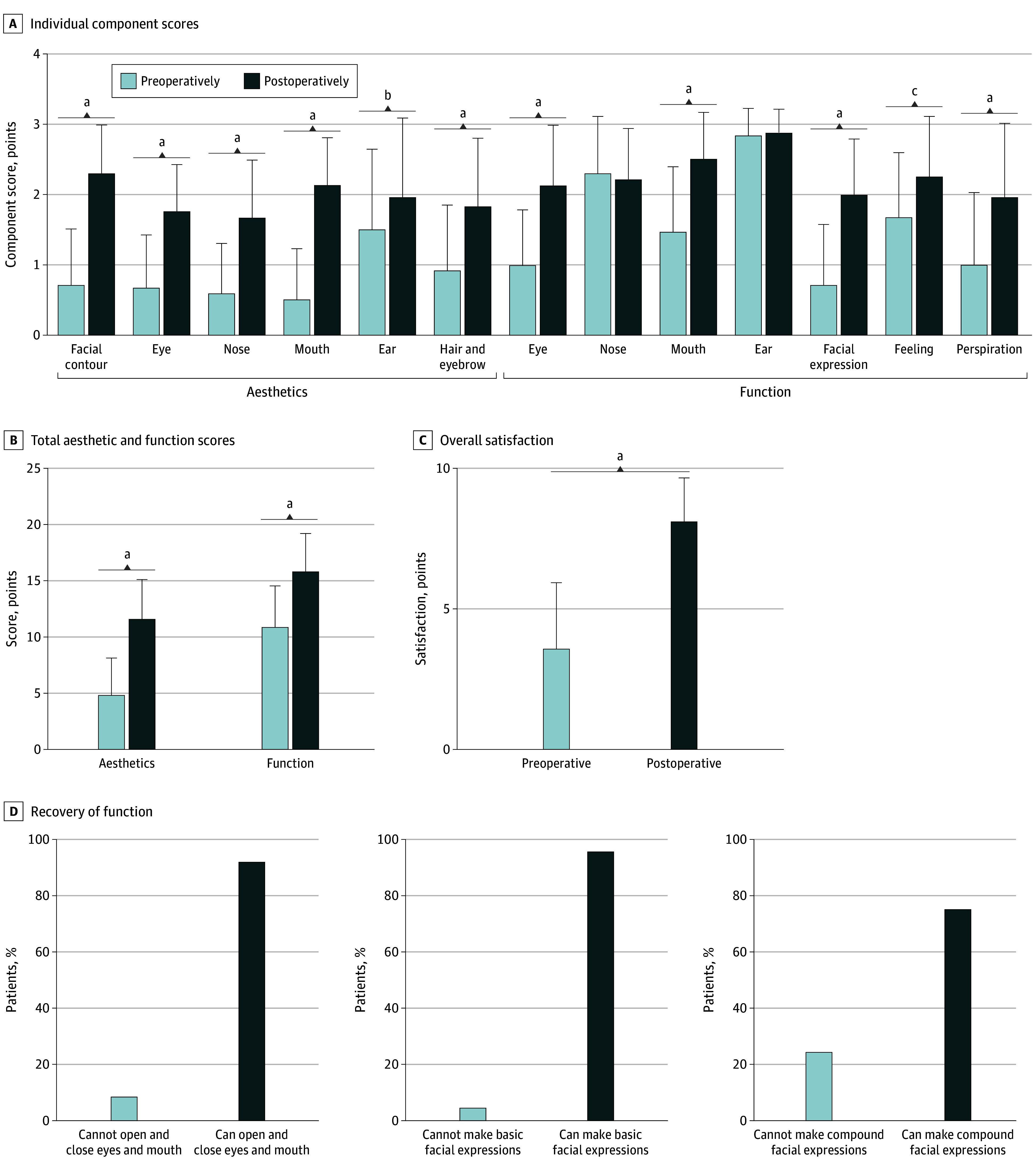
Aesthetic and Functional Status Score of Facial Soft-Tissue Deformities/Defects Scores A, Individual components of the Aesthetic and Functional Status Score of Facial Soft-Tissue Deformities/Defects scale preoperatively and postoperatively. B, Total aesthetic and functional scores preoperatively and postoperatively. C, Overall satisfaction with aesthetic and functional status preoperatively and postoperatively. D, Recovery of basic facial functions and expressions. Error bars indicate 95% CIs. ^a^*P* < .001. ^b^*P* < .01. ^c^*P* < .05.

Patients were followed up for a mean (range) of 5.6 (2-12) years. There was a significant improvement in aesthetic status in general (Figure 2). The greatest aesthetic improvement was found in mouth morphology. The overall mean (SD) aesthetic scores increased from 4.96 (3.26) of 18 points preoperatively to 11.52 (3.49) of 18 points postoperatively (Figure 2B).

#### Functional Outcome

A significant improvement in functional status in general was also noticed, except for ventilation and hearing functions (Figure 2). The most significant functional improvements were found in eye opening and closure. The mean (SD) functional scores increased from 11.09 (3.51) of 21 points preoperatively to 15.78 (3.26) of 21 points postoperatively (*P* < .001) (Figure 2B). The overall mean (SD) satisfaction of both aesthetic and functional status increased to 8.13 (1.52) of 10 points postoperatively compared with 3.58 (2.31) of 10 points preoperatively (*P* < .001) (Figure 2C).

Through photograph and video documentation, we found the grade 1 function was preserved in 22 of 24 patients (92%). A total of 23 patients (96%) achieved facial function of grade 2 (basic facial expression), and 18 (75%) achieved facial function of grade 3 (compound facial expression) (Figure 2D). There was a significant improvement in facial sensation. All patients developed sensations in their faces by the last follow-up visit. A total of 12 patients (50%) had full sensation recovery by the last follow-up visit compared with 5 (21%) preoperatively.

#### Quality of Life

**Figure 3.**
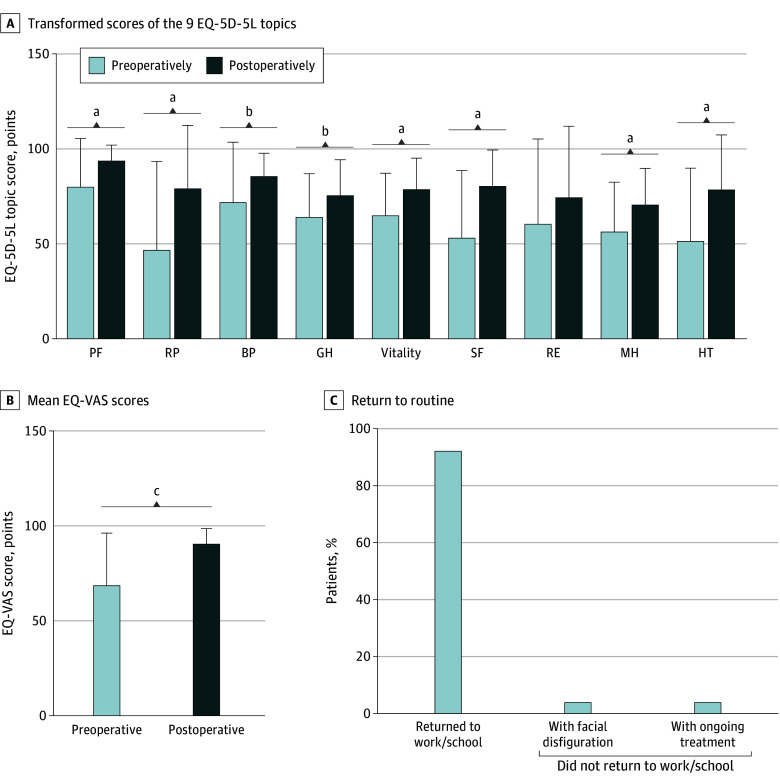
Quantitative Outcome Measures of Quality of Life by Questionnaires The questionnaires used included the 36-Item Short Form Health Survey and the EuroQoL Health-Related Quality of Life (EQ-5D-5L). A, The transformed result of the 9 topics of the Short Form Health Survey, including physical functioning (PF), role physical (RP), bodily pain (BP), general health (GH), vitality, social functioning (SF), role emotional (RE), mental health (MH), and reported health transition (HT), with higher scores indicating better quality of life. Significant improvement was achieved in all aspects postoperatively except for RE. B, EuroQol visual analog scale (EQ-VAS) revealing better self-reported health status postoperatively. C, The rate of patients who returned to work or school. A total of 22 of 24 patients (92%) were confident enough to go back to work or school after the procedure. Error bars indicate 95% CIs. ^a^*P* < .01. ^b^*P* < .05. ^c^*P* < .001.

Significant improvement in quality of life was indicated after the procedures compared with preoperatively according to SF-36 scores (Figure 3A). Notable improvements were observed in quality of life. The EuroQol visual analogue scale in EQ-5D-5L suggested a significantly improved self-reported health status, from a mean (SD) of 68.88 (27.19) preoperatively to 91.17 (7.85) postoperatively (*P* < .001) (Figure 3B). A total of 22 patients (92%) were back to work after the treatment (Figure 3C).

### Case Series

#### Case 1

**Figure 4.**
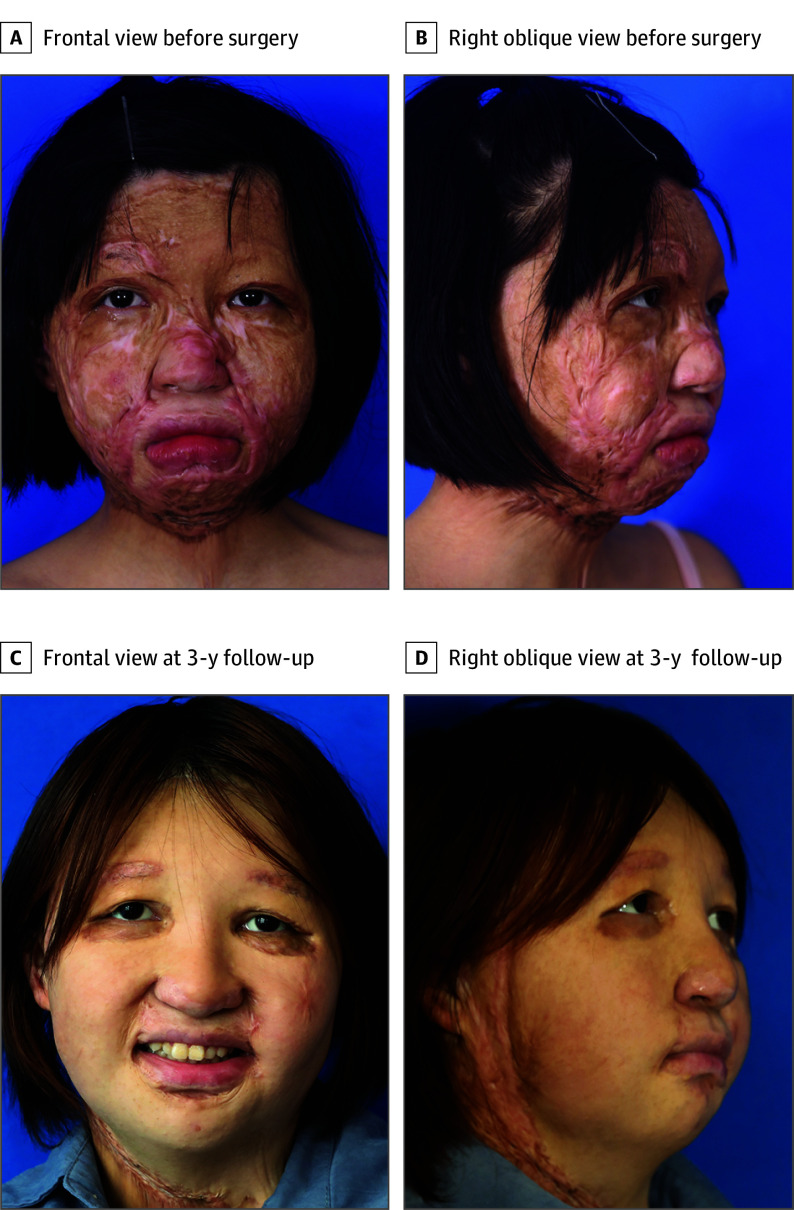
Clinical Images of Patient 1 Before Surgery and at 3-Year Follow-Up A and B, In patient 1, preoperative clinical photographs show total face deformities after a burn injury. B, At 3-year follow-up, the patient showed satisfactory facial appearance.

A 13-year-old girl had a total face disfigurement and severe restriction of facial expression after burn injury 1 year ago (Figure 4A and B). All her surgical procedures are summarized in a chronological manner in eFigure 2 in Supplement 1. In the first-stage surgery, vascular carrier transfer and tissue expander implantation were performed. Two years after the first-stage surgery, the total expander volume reached 2850 mL. A 38 × 30–cm^2^ prefabricated flap was obtained and transposed to the facial defect. The preserved internal mammary artery perforators and lateral thoracic arteries were anastomosed to the superficial temporal vessels for supercharging. At 3-year follow-up, the patient was satisfied with her new face, which had a uniform skin color and improved facial contour and nasal profile (Figure 4C and D). In addition to basic social expressions, including smiling and sadness, the patient was also able to make subtle facial expressions, including frowning and snarling. The restoration of facial expression at 3-year follow-up is shown in eFigure 3 in Supplement 1. Two months after her second-stage reconstruction, she was confident enough to return to her school as a student.

#### Case 2

**Figure 5.**
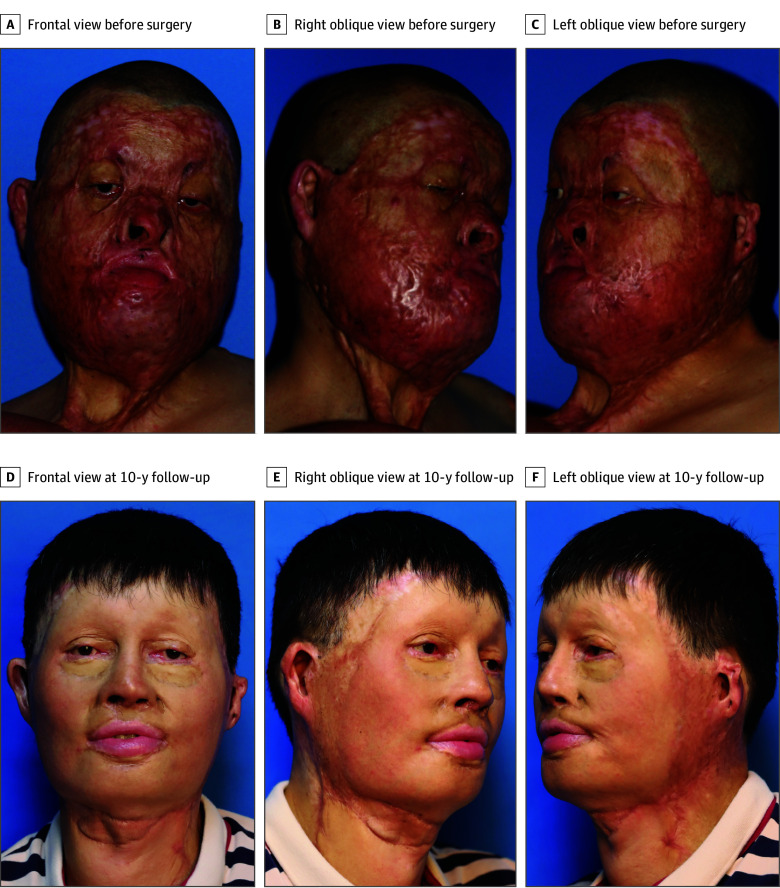
Clinical Images of Patient 2 Before Surgery and at 10-Year Follow-Up A-C, In patient 2, preoperative clinical photographs show total face deformities after a welding injury. D-F, At 10-year follow-up, the patient showed satisfactory facial appearance. This patient was first reported in our previous publication.^20^

A 41-year-old man with a totally disfigured face and severely restricted facial expression accepted the face prefabrication procedure. All his surgical procedures are summarized in a chronological manner in eFigure 2 in Supplement 1. The total expander volume reached 3200 mL. A 32 × 30–cm^2^ prefabricated flap was obtained and transposed to the facial defect. At 10-year follow-up, the patient was satisfied with his new face, which had a uniform skin color and improved facial contour and nasal profiles (Video). Functional deformities, including lagophthalmos, limited mouth opening, and limited neck range of motion, were greatly improved. Due to the soft skin texture of the new face, a remarkable improvement in facial expression was achieved. In addition to basic facial expressions, including smiling, laughing, anger, and pucker, the patient could also deliver delicate facial expressions, including frowning and wrinkling of the nose. The patient remained happy with his new face and was confident to return to work 1 year after the second procedure (Figure 5; eFigure 4 in Supplement 1).

**Video.** Facial Expressions of Case 2 at 10-Year Follow-Up After Face Reconstruction This video demonstrates the restoration of facial expressions, including eyebrow raising, nose wrinkling, eye closing, pouting, cheek puffing, teeth showing, smiling, and laughing, in case 2 at 10-year follow-up after facial reconstruction with the face prefabrication procedure.

## Discussion

Despite the long-standing history of autologous tissue in wound repair, its capacity in total face restoration is still deemed inferior and insufficient, as it frequently results in a patchlike appearance or new facial disfigurements. As a result, it is regarded as the last resort of wound coverage instead of a viable option in case of face disfigurement repair.

However, with the progress in science and modern surgical techniques, the capacity of autologous tissue in total face restoration has been extensively extended and serves as a viable option for the restoration of severe total face disfigurement. The primary challenges in total facial restoration using autologous tissue involve creating a flap of sufficient size to cover the entire face while also matching the face’s thickness and texture and accentuating facial features. Furthermore, ensuring the survival of this oversized flap, along with its long-term stability posttransfer, is crucial. It is hardly possible to simultaneously fulfill all these requirements with conventional methods. However, through the combination of modern technologies and up-to-date surgical techniques, we are now able to achieve these goals stably and repeatedly.^22^ The patients presented here, predominantly involving soft tissue and cartilage defects, extend beyond the capabilities of conventional reconstruction methods. With our novel approach, we successfully restored the total face disfigurement of our patients, leading to a significant enhancement in their quality of life. Although the cosmetic results may not match those of the best total face allotransplants, the achievement of a perceived normal appearance significantly enhances self-confidence and social esteem, evidenced by 22 of 24 patients (92%) returning to work or school. Encouragingly, the 5-year outcomes confirm the longevity and effectiveness of this technique. Interestingly, our results show improvement over time, likely due to scar softening and remodeling, enhancing facial expressions.

This result is achieved through the combination of a series of techniques, including flap prefabrication, skin expansion, 3-dimensional printing–based prelamination, supercharging, ICG–assisted monitored opening, and flap transfer. By summarizing the results of the 24 cases, we came up with a standardized and finalized approach consisting of 3 major sessions and multiple principle procedures (Figure 1), among which there are several key steps that distinguish this approach from the conventional methods and make the restoration of the total facial disfigurement possible.

### Prefabrication and Tissue Expansion Assisted Donor Tissue Preparation

The first key step toward total face restoration is to generate a donor skin large enough to cover the whole face, which is beyond the scope of conventional methods. There are 2 limitations that need to be conquered: safe and reliable perfusion, and a large donor tissue suitable for future facial coverage.

Restoring the entire face necessitates a large donor flap, which often cannot be adequately perfused by its native blood supply. To enhance perfusion, we preimplanted a vascular pedicle (composed of the descending branches of the lateral circumflex femoral vessels and surrounding fascia) under the chest skin prior to tissue expansion. If ischemia or venous congestion is observed during flap transfer, supercharging based on the second or third internal mammary perforators (due to their ample caliber for microsurgical anastomosis) can be used to further augment blood supply.^15,16,17,18,19,21,22,23^

As summarized in our previous publication,^20^ there are several characteristics that are indispensable for the donor tissue to properly reconstruct a face: the donor tissue should match in color and texture, be large enough to cover the defect, and be thin enough to accentuate facial features (summarized as the MLT properties). By combining flap prefabrication with skin and soft tissue expansion, we are able to consistently and reliably create a flap with the MLT properties for defect restoration without deviations in color and texture.

### One-Stage Fissure Opening Based on ICG–Assisted Monitoring

In our old version of total face restoration, the fissure opening was scheduled 4 weeks later after the flap transfer to ensure the safety of the donor tissue, which necessitated tracheotomy during this time span and greatly increased complications, such as inflammation. The advent of intraoperative IGA assists in creating openings for the eyes, nose, and mouth and successfully reduced this into a 1-stage procedure, which greatly reduced the risk of inflammation and voided the need for tracheostomy.

### 3-Dimensional Simulation-Assisted Flap Prelamination

This approach also extends to more complex cases involving middle and lower central facial defects, where composite tissue loss occurs. With 3-dimensional simulation-assisted flap prelamination and autologous costal cartilage grafts, missing composite tissue units can be recreated within the prefabricated flap to fit the patient’s facial contours.^23^

Our initial foray into facial restoration using autologous tissue dates back 17 years. We reported the successful restoration of type III and IV facial deformities in 42 patients.^15,16,17^ With accumulated experience, we recognized this technique’s potential in addressing total facial disfigurement,^15^ eventually leading to the consolidation and systematization of our fragmented experiences and surgical techniques into a standardized approach. This innovative approach differs from conventional methods in several key aspects: (1) it achieves total facial coverage with uniform color and texture similar to the original face; (2) it successfully accentuates or reconstructs facial features, such as the nose and lips; (3) it ensures safe flap transfer and reliable survival; (4) it minimizes the frequency of surgical procedures, thereby substantially reduced risks and patient suffering; and (5) it guarantees stable and long-term results. The success of our current approach in restoring total facial disfigurement is attributed to several key components: (1) enhancement of donor tissue vascularization using advanced prefabricated flap techniques, including vascular supercharging; (2) creation of an extremely large flap via skin and soft tissue expansion techniques; and (3) safe, reliable flap transfer with IGA-assisted monitoring of tissue perfusion. The limitation of the current approach lies in the reconstruction of the fine cosmetic details, including the eyelids and the lips. Future progress in tissue engineering or in vivo tissue engineering might hold a promise in providing a solution.

Our approach may benefit a substantial proportion of patients who are candidates for facial restoration. After analyzing the severity of preoperative injuries in patients requiring face transplant worldwide reported by Kantar et al,^24^ we found that approximately one-third to two-thirds of these patients with soft tissue or combined bony deformities could also be repaired with our approach. In conjunction with allograft face transplant, there are now 2 options for patients with significant facial disfigurement. One is face allotransplant, which provides near-normal facial features but is accompanied by life-long treatment of immunosuppression. The other is our approach as described in this report. This option currently will be less aesthetically perfect in recreating fine facial features, but there should be no further concerns related to immunosuppression.

### Limitations

This study has limitations. The limitations of this study are inherent to its retrospective nature and the absence of a control group. This is largely due to the complexity of the cases and the specialized nature of the procedures involved.

## Conclusions

The face prefabrication technique is particularly favorable for patients with soft tissue damage or loss, including facial skin and cartilage. Patients with more complex defects involving mimetic muscle or bone may still require allotransplant. However, with ongoing refinements in facial reanimation and bony reconstruction, our approach might soon become a viable option for these patients, providing acceptable outcomes without additional health concerns. The incorporation of both conventional and innovative approaches, such as flap prefabrication, 3-dimensional printing, and ICG-assisted monitoring, has significantly broadened the capabilities of autologous tissue reconstruction in facial restoration, yielding superior outcomes. This presents a valuable tool that may be effectively translated to other surgical contexts.

## Notes

Supplement 1.
eMethods.
**eAppendix 1.** 36-Item Short Form Health Survey questionnaire (SF-36) **eAppendix 2.** Aesthetic and Functional Status Score of Facial Soft-Tissue Deformities/Defects (A&F) **eAppendix 3.** EuroQol 5 Dimensions 5 Levels Questionnaire (EQ-5D-5L) **eFigure 1.** Surgical Procedures of Prefabrication **eFigure 2.** Chronological Description of Surgical Procedures and Events for Patients 1 and 2 **eFigure 3.** Restoration of Facial Expression at 3-Year Follow-Up in Patient 1 **eFigure 4.** Preoperative and Postoperative Photographs of Patient 2

Supplement 2. Data Sharing Statement

## References

[1] Soni CV, Barker JH, Pushpakumar SB, . Psychosocial considerations in facial transplantation. Burns. 2010;36(7):959-964. doi:10.1016/j.burns.2010.01.012 20378255

[2] Ozmen S, Uygur S, Eryilmaz T, Ak B. Facial resurfacing with a monoblock full-thickness skin graft after multiple malignant melanomas excision in xeroderma pigmentosum. J Craniofac Surg. 2012;23(5):1542-1543. doi:10.1097/SCS.0b013e31824e660e 22976654

[3] Angrigiani C, Grilli D. Total face reconstruction with one free flap. Plast Reconstr Surg. 1997;99(6):1566-1575. doi:10.1097/00006534-199705010-00014 9145124

[4] Teot L, Cherenfant E, Otman S, Giovannini UM. Prefabricated vascularised supraclavicular flaps for face resurfacing after postburns scarring. Lancet. 2000;355(9216):1695-1696. doi:10.1016/S0140-6736(00)02245-5 10905249

[5] Pribaz JJ, Caterson EJ. Evolution and limitations of conventional autologous reconstruction of the head and neck. J Craniofac Surg. 2013;24(1):99-107. doi:10.1097/SCS.0b013e31827104ab 23348264

[6] Devauchelle B, Badet L, Lengelé B, . First human face allograft: early report. Lancet. 2006;368(9531):203-209. doi:10.1016/S0140-6736(06)68935-6 16844489

[7] Lantieri L, Cholley B, Lemogne C, . First human facial retransplantation: 30-month follow-up. Lancet. 2020;396(10264):1758-1765. doi:10.1016/S0140-6736(20)32438-7 33248497

[8] Lantieri L, Grimbert P, Ortonne N, . Face transplant: long-term follow-up and results of a prospective open study. Lancet. 2016;388(10052):1398-1407. doi:10.1016/S0140-6736(16)31138-2 27567680

[9] Rifkin WJ, David JA, Plana NM, . Achievements and challenges in facial transplantation. Ann Surg. 2018;268(2):260-270. doi:10.1097/SLA.0000000000002723 29489486

[10] Tasigiorgos S, Krezdorn N, Bueno E, Pomahac B. New avenues of vascularized composite allotransplantation and their potential risks and benefits. Ann Surg. 2017;266(2):e25. doi:10.1097/SLA.0000000000002294 28525412

[11] Kiwanuka H, Bueno EM, Diaz-Siso JR, Sisk GC, Lehmann LS, Pomahac B. Evolution of ethical debate on face transplantation. Plast Reconstr Surg. 2013;132(6):1558-1568. doi:10.1097/PRS.0b013e3182a97e2b 24281583

[12] Siemionow M. The past the present and the future of face transplantation. Curr Opin Organ Transplant. 2020;25(6):568-575. doi:10.1097/MOT.0000000000000812 33044347

[13] Tasigiorgos S, Kollar B, Krezdorn N, Bueno EM, Tullius SG, Pomahac B. Face transplantation-current status and future developments. Transpl Int. 2018;31(7):677-688. doi:10.1111/tri.13130 29421860

[14] Krezdorn N, Lian CG, Wells M, . Chronic rejection of human face allografts. Am J Transplant. 2019;19(4):1168-1177. doi:10.1111/ajt.15143 30312535 PMC6433509

[15] Zan T, Gao Y, Li H, Gu B, Xie F, Li Q. Pre-expanded, prefabricated monoblock perforator flap for total facial resurfacing. Clin Plast Surg. 2017;44(1):163-170. doi:10.1016/j.cps.2016.08.007 27894577

[16] Zhou SB, Zhang GY, Xie Y, . Autologous stem cell transplantation promotes mechanical stretch induced skin regeneration: a randomized phase I/II clinical trial. EBioMedicine. 2016;13:356-364. doi:10.1016/j.ebiom.2016.09.031 27876353 PMC5264315

[17] Li QF, Zan T, Li H, . Reconstruction of postburn full facial deformities with an integrated method. J Craniofac Surg. 2016;27(5):1175-1180. doi:10.1097/SCS.0000000000002800 27307306

[18] Li Q, Zan T, Gu B, . Face resurfacing using a cervicothoracic skin flap prefabricated by lateral thigh fascial flap and tissue expander. Microsurgery. 2009;29(7):515-523. doi:10.1002/micr.20640 19308953

[19] Zan T, Li H, Gu B, . Surgical treatment of facial soft-tissue deformities in postburn patients: a proposed classification based on a retrospective study. Plast Reconstr Surg. 2013;132(6):1001e-1014e. doi:10.1097/PRS.0b013e3182a97e81 24281605

[20] Li Q, Zan T, Li H, . Flap prefabrication and stem cell-assisted tissue expansion: how we acquire a monoblock flap for full face resurfacing. J Craniofac Surg. 2014;25(1):21-25. doi:10.1097/01.scs.0000436743.75289.6b 24406553

[21] Li H, Gao Y, Gu B, . Midface prelamination by using a three-dimensional cervicothoracic prefabricated flap. Plast Reconstr Surg. Published online June 22, 2023. doi:10.1097/PRS.0000000000010882 37344938

[22] Huang X, Li H, Gu S, . Intraoperative indocyanine green angiography facilitates flap fenestration and facial organ fabrication in total facial restoration. Plast Reconstr Surg. Published online June 28, 2023. doi:10.1097/PRS.0000000000010891 37382913 PMC11104494

[23] Zan T, Li H, Huang X, . Augmentation of perforator flap blood supply with sole or combined vascular supercharge and flap prefabrication for difficult head and neck reconstruction. Facial Plast Surg Aesthet Med. 2020;22(6):441-448. doi:10.1089/fpsam.2020.0040 32668181

[24] Kantar RS, Alfonso AR, Diep GK, . Facial transplantation: principles and evolving concepts. Plast Reconstr Surg. 2021;147(6):1022e-1038e. doi:10.1097/PRS.0000000000007932 34019516

