# Supplementary material for: Autologous Tissue Repair and Total Face Restoration
Source: JAMA Otolaryngol Head Neck Surg. 2024 Jul 3;150(8):695–703. doi: 10.1001/jamaoto.2024.1572 (PMC11310821; doi:10.1001/jamaoto.2024.1572)
Supplement: Supplement 1. — eMethods. eAppendix 1. 36-Item Short Form Health Survey questionnaire (SF-36) eAppendix 2. Aesthetic and Functional Status Score of Facial Soft-Tissue Deformities/Defects (A&F) eAppendix 3. EuroQol 5 Dimensions 5 Levels Questionnaire (EQ-5D-5L) eFigure 1. Surgical Procedures of Prefabrication eFigure 2. Chronological Description of Surgical Procedures and Events for Patients 1 and 2 eFigure 3. Restoration of Facial Expression at 3-Year Follow-Up in Patient 1 eFigure 4. Preoperative and Postoperative Photographs of Patient 2 [file jamaotolaryngolheadnecksurg-e241572-s001.pdf]

Supplemental Online Content

Zan T, Wang W, Li H, et al. Autologous tissue repair and total face restoration. *JAMA Otolaryngol Head Neck Surg*. Published online July 3, 2024.
doi:10.1001/jamaoto.2024.1572

eMethods.

eAppendix 1. 36-Item Short Form Health Survey questionnaire (SF-36)

eAppendix 2. Aesthetic and Functional Status Score of Facial Soft-Tissue Deformities/Defects (A&F)

eAppendix 3. EuroQol 5 Dimensions 5 Levels Questionnaire (EQ-5D-5L)

eFigure 1. Surgical Procedures of Prefabrication

eFigure 2. Chronological Description of Surgical Procedures and Events for Patients 1 and 2

eFigure 3. Restoration of Facial Expression at 3-Year Follow-Up in Patient 1

eFigure 4. Preoperative and Postoperative Photographs of Patient 2

This supplementary material has been provided by the authors to give readers additional information about their work.

© 2024 Zan T et al. *JAMA Otolaryngology–Head & Neck Surgery.*

eMethods.

1. Preoperative Planning and Donor Site Selection

We utilized 3-Dimensional planning as part of the preoperative workup. Based on the results of computed tomography, the “new” faces were simulated with use of the Mimics CAD/CAM software (Materialise, Ann Arbor, Michigan, USA). We also used 3D analysis to estimate the area of the anterior thoracic skin needed to resurface the given facial defects. Donor site selection was based on the fundamental principle of “replacing like with like.” Due to the scarcity of well-preserved skin in burn patients, we have proposed a donor site selection protocol for face prefabrication. Skin donors from the supraclavicular area, the anterior chest region, and the lateral thoracic area are the preferred donor sites over all other sites of the body.

2. Harvest and Transfer of Vascular Carrier

The full length of the descending branch of the lateral circumflex femoral artery, vein and surrounding fascia were harvested as a free fascial flap that served as a vascular carrier. The pedicle vessels were anastomosed to the superior thyroid artery and vein. This well vascularized free fascial flap from the anterolateral thigh was then placed in the subcutaneous pocket beneath the skin of the anterior chest wall. The flap itself augmented the blood supply to the cutaneous tissues above and ensured adequate vascularity after tissue expansion. A rectangular tissue expander was then placed underneath the fascial flap for future expansion.

3. Skin and soft-tissue Expansion

Tissue expansion began one week post-operatively. The expander was inflated with physiological saline twice weekly until the expanded cutaneous flap exceeded 120% of the area of the calculated facial defect. In general, the expanded skin on the anterior chest wall could become extremely thin or the surface area of the expanded skin might not be large enough to cover the entire face.

4. Transfer of the Prefabricated “New” Face after Assessment of Its Perfusion

We utilized a two-team approach in the operating room during the final stage of full facial reconstruction. One team elevated the donor skin flap from the anterior chest wall nourished by the prefabricated vessels while reserving the internal mammary artery perforator (IMAP) and lateral thoracic perforator (LTA) for potential vascular supercharge. The donor site was closed with either a local flap or skin graft. The second team performed recipient site scar tissue excision, repositioned the deformed anatomic units and transferred the prefabricated skin flap and inset the flap to cover the entire facial defect to form a "new face".

Before the transfer of the prefabricated skin flap, doppler ultrasonography was performed to confirm the existence of the internal mammary vessels, the lateral thoracic vessels, as well as the pedicle of the prefabricated skin flap in order to decide whether to proceed with the transfer or to perform a two-week surgical delay if those vessels were found to be not reliable. Intraoperative indocyanine green angiography (IGA) was

© 2024 Zan T et al. *JAMA Otolaryngology–Head & Neck Surgery.*

performed with the SPY imaging system (Novadaq Technologies, Inc., Canada) to assist in the design of a multiple-pedicled, prefabricated, "new" face and to guide the design for subsequent openings for the mouth, nasal nostrils, and palpebral fissures. Briefly, a 2 mL bolus of ICG (2.5mg/mL; Dandong Yichuang Pharmaceutical Co., China) was injected through a peripheral intravenous line. The fluorescence detector was placed 20 cm above the skin surface to video record the entire course of the flap perfusion. [16] Before the transfer of prefabricated skin flap, perfusion territories of the prefabricated vessels, IMAP and LTA were evaluated one by one by clamping the other two pedicles. If the flap was observed to have hypo-perfused dark areas during maximal perfusion or with compromised venous outflow, additional pedicles were opened with repeated IGA to examine the skin flap perfusion. If the hypo-perfused area(s) still existed when all available pedicles were opened, excision of such poorly perfused tissue would be performed. The prefabricated flap was then transferred from the anterior chest to the face. According to ICGA evaluation, IMAP, LTA, or both were also dissected and included in the flap, and anastomosed to the superficial temporal vessels for vascular supercharge. The opening of nostrils, oral and palpebral orifices, together with organ fabrication, were conducted under the guidance of intraoperative IGA via hemodynamic evaluation of flap perfusion.

5. Reconstruction of Specific Facial Features

If any nasal, upper lip, or alveolar bony defects were required for reconstructions, the missing composite units were prelaminated within the donor skin site by insetting of autologous costal cartilage grafts. Guided by 3D-printed models, the cartilage framework was trimmed carefully to fit the bony outlines and soft-tissue profiles of the missing structures. For upper eyelid reconstruction, the original skin or scar tissue below the eyelid crease was preserved to avoid a bulky eyelid and allowed for subsequent formation of natural double eyelid. Sufficient skin flap preservation and secondary capsulectomy were essential for the reconstruction of natural-looking eyelids.

6. Secondary Revision

Periorbital revision and rhinoplasty were performed three weeks after the prefabricated "new" face transfer. Procedures such as Z-plasty, canthoplasty or nasal reconstruction could also be performed if indicated. Debulking, including removal of bulky pedicle tissues in the forehead, bilateral cheeks, and nasal and perioral areas was delayed for up to three weeks or longer after rhinoplasty.

7. Follow-up assessments

Three questionnaires, including the 36-Item Short Form Health Survey questionnaire (SF-36), Aesthetic and Functional Status Score of Facial Soft-Tissue Deformities/Defects (A&F) and Euroqol 5 dimensions 5 levels questionnaire (EQ-5D-5L), were assessed based on preoperative and postoperative situations respectively.

The raw scale scores of SF-36 in 9 aspects, including physical functioning (PF), role-physical (RP), bodily pain (BP), general health (GH), vitality (VT), social functioning (SF), role-emotional (RE), mental health (MH) and reported health transition (HT),

were calculated and transformed to a 0-100 scale respectively according to its manual and interpretation guide. In A&F, each aspect was valued from 0 to 3, and the scores of aesthetic and functional statuses were summed up (full marks: 18 points for the aesthetic status and 21 points for the functional status) respectively for further analysis.

© 2024 Zan T et al. *JAMA Otolaryngology–Head & Neck Surgery*.

eAppendix 1. 36-Item Short Form Health Survey questionnaire (SF-36)

GENERAL HEALTH:

In general, would you say your health is:

(1) Excellent (2) Very Good (3) Good (4) Fair (5) Poor

Compared to one year ago, how would you rate your health in general now?

(1) Much better now than one year ago

(2) Somewhat better now than one year ago

(3) About the same

(4) Somewhat worse now than one year ago

(5) Much worse than one year ago

LIMITATIONS OF ACTIVITIES:

The following items are about activities you might do during a typical day. Does your health now limit you in these activities? If so, how much?

Vigorous activities, such as running, lifting heavy objects, participating in strenuous sports.

(1) Yes, Limited a lot (2) Yes, Limited a Little (3) No, Not Limited at all

Moderate activities, such as moving a table, pushing a vacuum cleaner, bowling, or playing golf

(1) Yes, Limited a lot (2) Yes, Limited a Little (3) No, Not Limited at all

Lifting or carrying groceries

(1) Yes, Limited a lot (2) Yes, Limited a Little (3) No, Not Limited at all

Climbing several flights of stairs

(1) Yes, Limited a Lot (2) Yes, Limited a Little (3) No, Not Limited at all

Climbing one flight of stairs

(1) Yes, Limited a Lot (2) Yes, Limited a Little (3) No, Not Limited at all

Bending, kneeling, or stooping

(1) Yes, Limited a Lot (2) Yes, Limited a Little (3) No, Not Limited at all

© 2024 Zan T et al. *JAMA Otolaryngology–Head & Neck Surgery.*

Walking more than a mile

(1) Yes, Limited a Lot (2) Yes, Limited a Little (3) No, Not Limited at all

Walking several blocks

(1) Yes, Limited a Lot (2) Yes, Limited a Little (3) No, Not Limited at all

Walking one block

(1) Yes, Limited a Lot (2) Yes, Limited a Little (3) No, Not Limited at all

Bathing or dressing yourself

(1) Yes, Limited a Lot (2) Yes, Limited a Little (3) No, Not Limited at all

PHYSICAL HEALTH PROBLEMS:

During the past 4 weeks, have you had any of the following problems with your work or other regular daily activities as a result of your physical health?

Cut down the amount of time you spent on work or other activities

(1) Yes (2) No

Accomplished less than you would like

(1) Yes (2) No

Were limited in the kind of work or other activities

(1) Yes (2) No

Had difficulty performing the work or other activities (for example, it took extra effort)

(1) Yes (2) No

EMOTIONAL HEALTH PROBLEMS:

During the past 4 weeks, have you had any of the following problems with your work or other regular daily activities as a result of any emotional problems (such as feeling depressed or anxious)?

Cut down the amount of time you spent on work or other activities
(1) Yes (2) No

Accomplished less than you would like
(1) Yes (2) No

© 2024 Zan T et al. *JAMA Otolaryngology–Head & Neck Surgery.*

Didn't do work or other activities as carefully as usual
(1) Yes (2) No

SOCIAL ACTIVITIES:

Emotional problems interfered with your normal social activities with family, friends, neighbors, or groups?

(1) Not at all (2) Slightly (3) Moderately (4)Severe (5)Very Severe

PAIN:

How much bodily pain have you had during the past 4 weeks?

(1) None (2) Very Mild (3) Mild (4) Moderate (5)Severe (6)Very Severe

During the past 4 weeks, how much did pain interfere with your normal work (including both work outside the home and housework)?

(1) Not at all (2) A little bit (3) Moderately (4) Quite a bit (5) Extremely

ENERGY AND EMOTIONS:

These questions are about how you feel and how things have been with you during the last 4 weeks. For each question, please give the answer that comes closest to the way you have been feeling.

Did you feel full of pep?
(1) All of the time

(2) Most of the time

(3) A good bit of the time

(4) Some of the time

(5) A little bit of the time

(6) None of the time

Have you been a very nervous person?
(1) All of the time

(2) Most of the time

(3) A good bit of the time

(4) Some of the time

© 2024 Zan T et al. *JAMA Otolaryngology–Head & Neck Surgery.*

(5) A little bit of the time

(6) None of the time

Have you felt so down in the dumps that nothing could cheer you up?
(1) All of the time

(2) Most of the time

(3) A good bit of the time

(4) Some of the time

(5) A little bit of the time
(6) None of the time

Have you felt calm and peaceful?
(1) All of the time

(2) Most of the time

(3) A good bit of the time

(4) Some of the time

(5) A little bit of the time

(6) None of the time

Did you have a lot of energy?
(1) All of the time

(2) Most of the time

(3) A good bit of the time

(4) Some of the time

(5) A little bit of the time
(6) None of the time

Have you felt downhearted and blue?
(1) All of the time

(2) Most of the time

(3) A good bit of the time

(4) Some of the time

(5) A little bit of the time
(6) None of the time

© 2024 Zan T et al. *JAMA Otolaryngology–Head & Neck Surgery*.

Did you feel worn out?
(1) All of the time

(2) Most of the time

(3) A good bit of the time

(4) Some of the time

(5) A little bit of the time

(6) None of the time

Have you been a happy person?
(1) All of the time

(2) Most of the time

(3) A good bit of the time

(4) Some of the time

(5) A little bit of the time

(6) None of the time

Did you feel tired?
(1) All of the time

(2) Most of the time

(3) A good bit of the time

(4) Some of the time

(5) A little bit of the time

(6) None of the time

SOCIAL ACTIVITIES:
During the past 4 weeks, how much of the time has your physical health or emotional problems interfered with your social activities (like visiting with friends, relatives, etc.)?

(1) All of the time

(2) Most of the time

(3) Some of the time

(4) A little bit of the time

(5) None of the Time

© 2024 Zan T et al. *JAMA Otolaryngology–Head & Neck Surgery.*

GENERAL HEALTH:
How true or false is each of the following statements for you?

I seem to get sick a little easier than other people
(1) Definitely true (2) Mostly true (3) Don't know (4) Mostly false
(5)Definitely false

I am as healthy as anybody I know
(1) Definitely true (2) Mostly true (3) Don't know (4) Mostly false
(5)Definitely false

I expect my health to get worse
(1) Definitely true (2) Mostly true (3) Don't know (4) Mostly false
(5)Definitely false

My health is excellent
(1) Definitely true (2) Mostly true (3) Don't know (4) Mostly false
(5)Definitely false

© 2024 Zan T et al. *JAMA Otolaryngology–Head & Neck Surgery.*

eAppendix 2. Aesthetic and Functional Status Score of Facial Soft-Tissue Deformities/Defects (A&F)

Aesthetic Status	Facial contour	Satisfied（3 points）	
		Nearly satisfied（2 points）	
		Not satisfied but acceptable（1 point）	
		Not satisfied and not acceptable（0 point）	
	Eye	satisfied（3 points）	
		Nearly satisfied（2 points）	
		Not satisfied but acceptable（1 point）	
		Not satisfied and not acceptable（0 point）	
	Nose	Satisfied（3 points）	
		Nearly satisfied（2 points）	
		Not satisfied but acceptable（1 point）	
		Not satisfied and not acceptable（0 point）	
	Mouth	Satisfied（3 points）	
		Nearly satisfied（2 points）	
		Not satisfied but acceptable（1 point）	
		Not satisfied and not acceptable（0 point）	

© 2024 Zan T et al. *JAMA Otolaryngology–Head & Neck Surgery.*

	Ear	Satisfied（3 points）	
		Nearly satisfied（2 points）	
		Not satisfied but acceptable（1 point）	
		Not satisfied and not acceptable（0 point）	
	Hair and Eyebrow	Satisfied（3 points）	
		Nearly satisfied（2 points）	
		Not satisfied but acceptable（1 point）	
		Not satisfied and not acceptable（0 point）	
Functional Status	Eye	Eyelids can be closed naturally（3 points）	
		Eyelid can be closed with effort（2 points）	
		Eyelid cannot be fully closed（1 point）	
		Eyeball exposure（0 point）	
	Nose	Unobstructed ventilation（3 points）	
		Slightly obstructed ventilation（2 points）	
		Obviously obstructed ventilation（1 point）	
		Completely blocked ventilation（0 point）	
	Mouth	Can normally open and close mouth, eat and speak（3 points）	

© 2024 Zan T et al. *JAMA Otolaryngology–Head & Neck Surgery.*

		Have problems with opening and closing mouth, eating or speaking, but daily life is not affected（2 points）	
		Have problems with opening and closing mouth, eating or speaking, and daily life is affected（1 point）	
		Totally cannot open and close mouth, eat or speak（0 point）	
	Ear	Normal hearing（3 points）	
		Hearing is slightly impaired, but daily life is not affected（2 points）	
		Hearing is impaired, and daily life is affected（1 point）	
		Hearing loss（0 point）	
	Facial expressions	Can naturally make facial expressions（3 points）	
		Can make facial expressions, but expressions are slightly unnatural（2 points）	
		Can make facial expressions, but expressions are unnatural（1 point）	
		Cannot make facial expressions（0 point）	
	Feeling (Injured	Can feel hot and cold, pain and touch（3 points）	

© 2024 Zan T et al. *JAMA Otolaryngology–Head & Neck Surgery.*

	/ surgical site)	Can feel hot and cold, pain and touch, but the sensation slightly decreases (2 points)	
		Can feel hot and cold, pain and touch, but the sensation significantly decreases (1 point)	
		Cannot feel hot and cold, pain and touch (0 point)	
	Perspiration (Injured / surgical site)	Perspiration is completely normal (3 points)	
		Perspiration is a little hard (2 points)	
		Perspiration is very hard (1 point)	
		Cannot perspire at all (0 point)	
Satisfaction (0 to 10 points, with 10 points as a full mark)			

© 2024 Zan T et al. *JAMA Otolaryngology–Head & Neck Surgery*.

eAppendix 3. EuroQol 5 Dimensions 5 Levels Questionnaire (EQ-5D-5L)

Under each heading, please tick the ONE box that best describes your health TODAY.

MOBILITY

I have no problems in walking about □

I have slight problems in walking about □

I have moderate problems in walking about □

I have severe problems in walking about □

I am unable to walk about □

SELF-CARE

I have no problems washing or dressing myself □

I have slight problems washing or dressing myself □

I have moderate problems washing or dressing myself □

I have severe problems washing or dressing myself □

I am unable to wash or dress myself □

USUAL ACTIVITIES (*e.g. work, study, housework, family or leisure activities*)

I have no problems doing my usual activities □

I have slight problems doing my usual activities □

I have moderate problems doing my usual activities □

I have severe problems doing my usual activities □

I am unable to do my usual activities □

PAIN/DISCOMFORT

I have no pain or discomfort □

I have slight pain or discomfort □

I have moderate pain or discomfort □

I have severe pain or discomfort □

© 2024 Zan T et al. *JAMA Otolaryngology–Head & Neck Surgery.*

I have extreme pain or discomfort □

ANXIETY/DEPRESSION

I am not anxious or depressed □

I am slightly anxious or depressed □

I am moderately anxious or depressed □

I am severely anxious or depressed □

I am extremely anxious or depressed □

- We would like to know how good or bad your health is TODAY.
- This scale is numbered from 0 to 100.
- 100 means the best health you can imagine.
- 0 means the worst health you can imagine.
- Mark an X on the scale to indicate how your health is TODAY.
- Now, please write the number you marked on the scale in the box below.

YOUR HEALTH TODAY= ☐

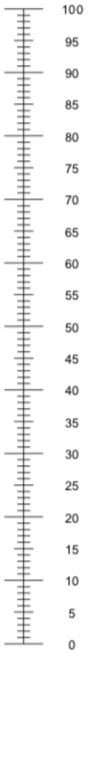

© 2024 Zan T et al. *JAMA Otolaryngology–Head & Neck Surgery.*

eFigure 1. Surgical Procedures of Prefabrication

A. The descending branch of the lateral circumflex femoral vessels and surrounding fascia were harvested as a vascular carrier form the thigh. B. The vascular carrier was anastomosed with the facial vessels in this case. C. The fascia of the vascular carrier was placed and secured in the subcutaneous pocket of the donor site over the tissue expander.

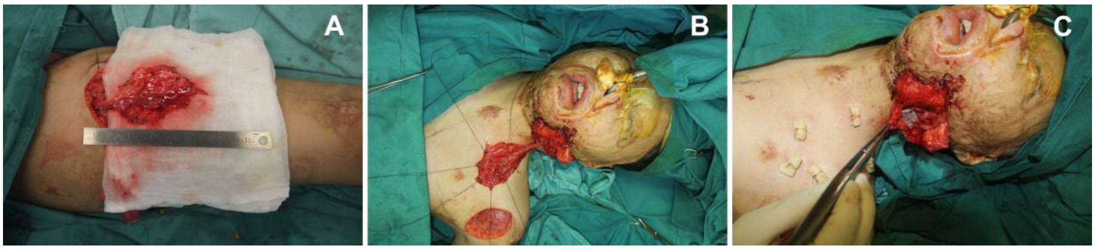

© 2024 Zan T et al. *JAMA Otolaryngology–Head & Neck Surgery.*

eFigure 2. Chronological Description of Surgical Procedures and Events for Patients 1 and 2

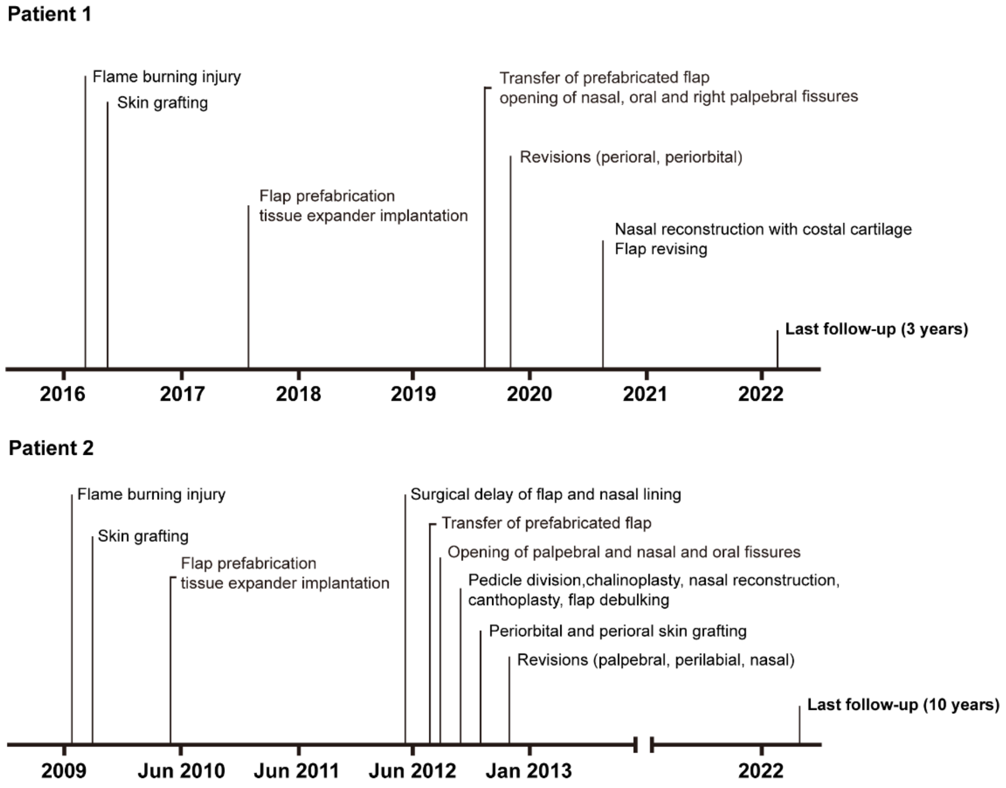

© 2024 Zan T et al. *JAMA Otolaryngology–Head & Neck Surgery*.

eFigure 3. Restoration of Facial Expression at 3-Year Follow-Up in Patient 1

Normal (Panel A), eyebrow raising (Panel B), nose wrinkling (Panel C), eye closing (Panel D), pouting (Panel E), cheek puffing (Panel F), teeth showing (Panel G), smiling (Panel H), and Laughing (Panel I).

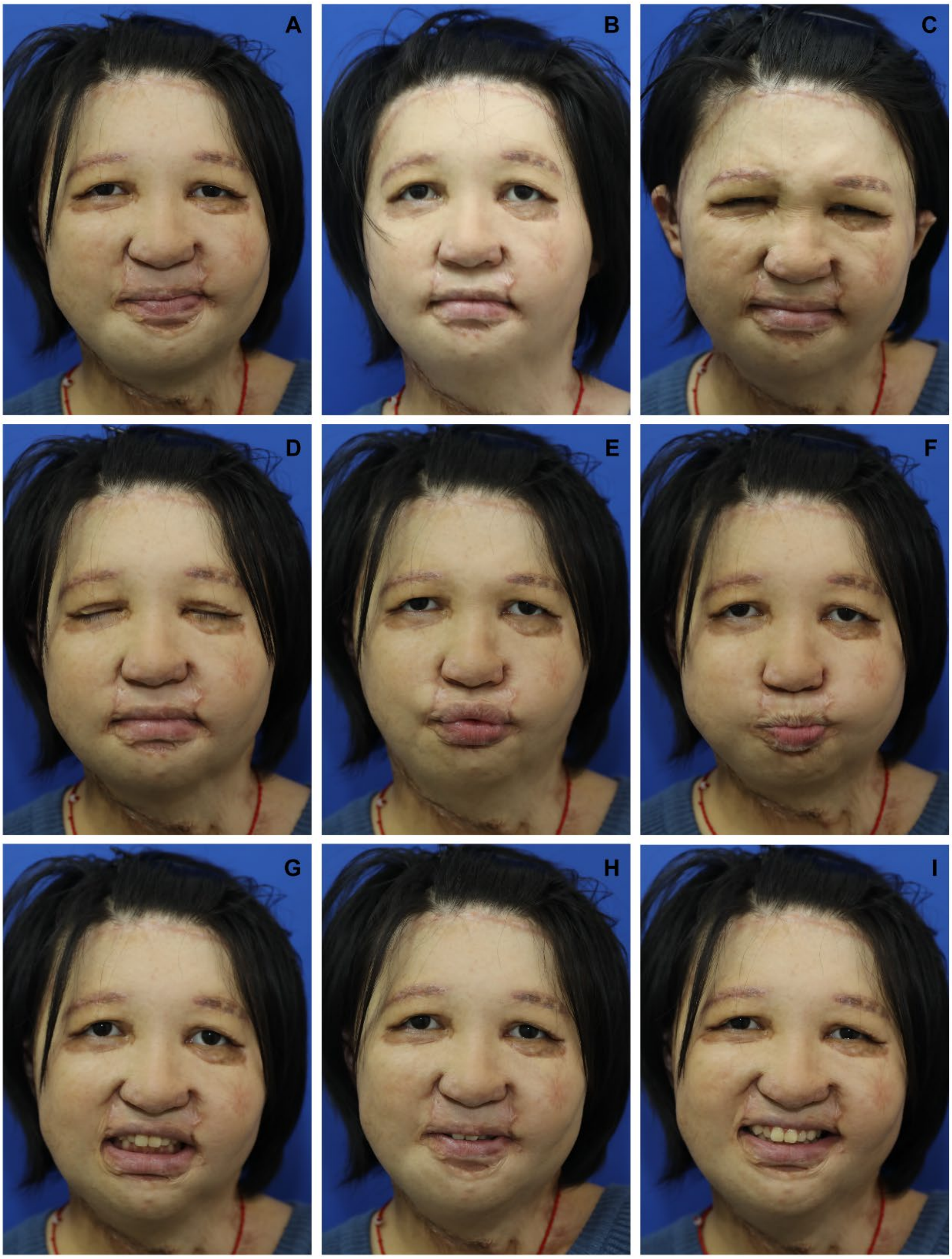

© 2024 Zan T et al. *JAMA Otolaryngology–Head & Neck Surgery.*

eFigure 4. Preoperative and Postoperative Photographs of Patient 2

Shown is the preoperative appearance of patient 1 (Panel A), and appearance after surgery at 1-year (Panel B), 5-year (Panel C) and 7-year (Panel D).

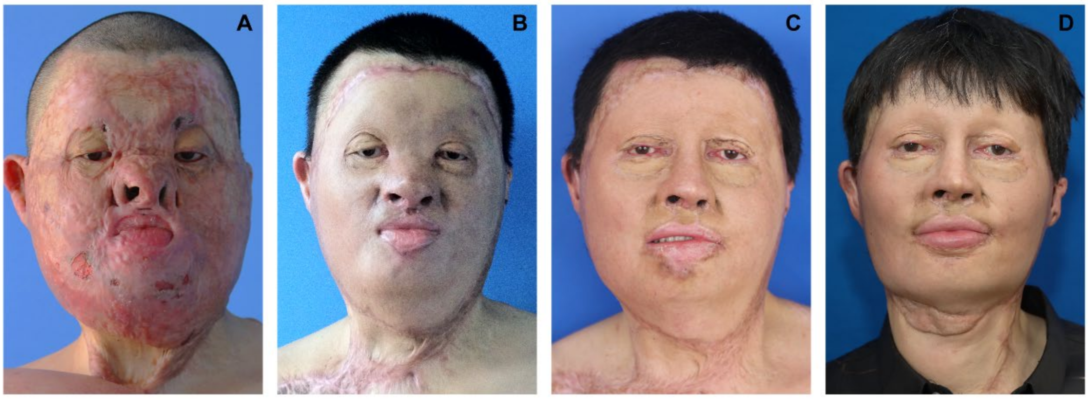

© 2024 Zan T et al. *JAMA Otolaryngology–Head & Neck Surgery.*
