# Supplementary material for: Autologous Tissue Repair and Total Face Restoration
Source: JAMA Otolaryngol Head Neck Surg. 2024 Jul 3;150(8):695–703. doi: 10.1001/jamaoto.2024.1572 (PMC11310821; doi:10.1001/jamaoto.2024.1572)
Supplement: Supplement 2. — Data Sharing Statement [file jamaotolaryngolheadnecksurg-e241572-s002.pdf]

Data Sharing Statement

Zan. Autologous Tissue Repair and Total Face Restoration. *JAMA Otolaryngol Head Neck Surg*. Published July 03, 2024. doi:10.1001/jamaoto.2024.1572

Data
Data available: No
